# Supplementary material for: Causal Relationship between Aging and Anorexia Nervosa: A White-Matter-Microstructure-Mediated Mendelian Randomization Analysis
Source: Biomedicines. 2024 Aug 16;12(8):1874. doi: 10.3390/biomedicines12081874 (PMC11351342; doi:10.3390/biomedicines12081874)
Supplement: Supplementary file 1 [file biomedicines-12-01874-s001.zip › Supplementary Figure/Figure S9/Leaveoneout plot of MR analysis for ubm-b-1942 to AN.pdf]

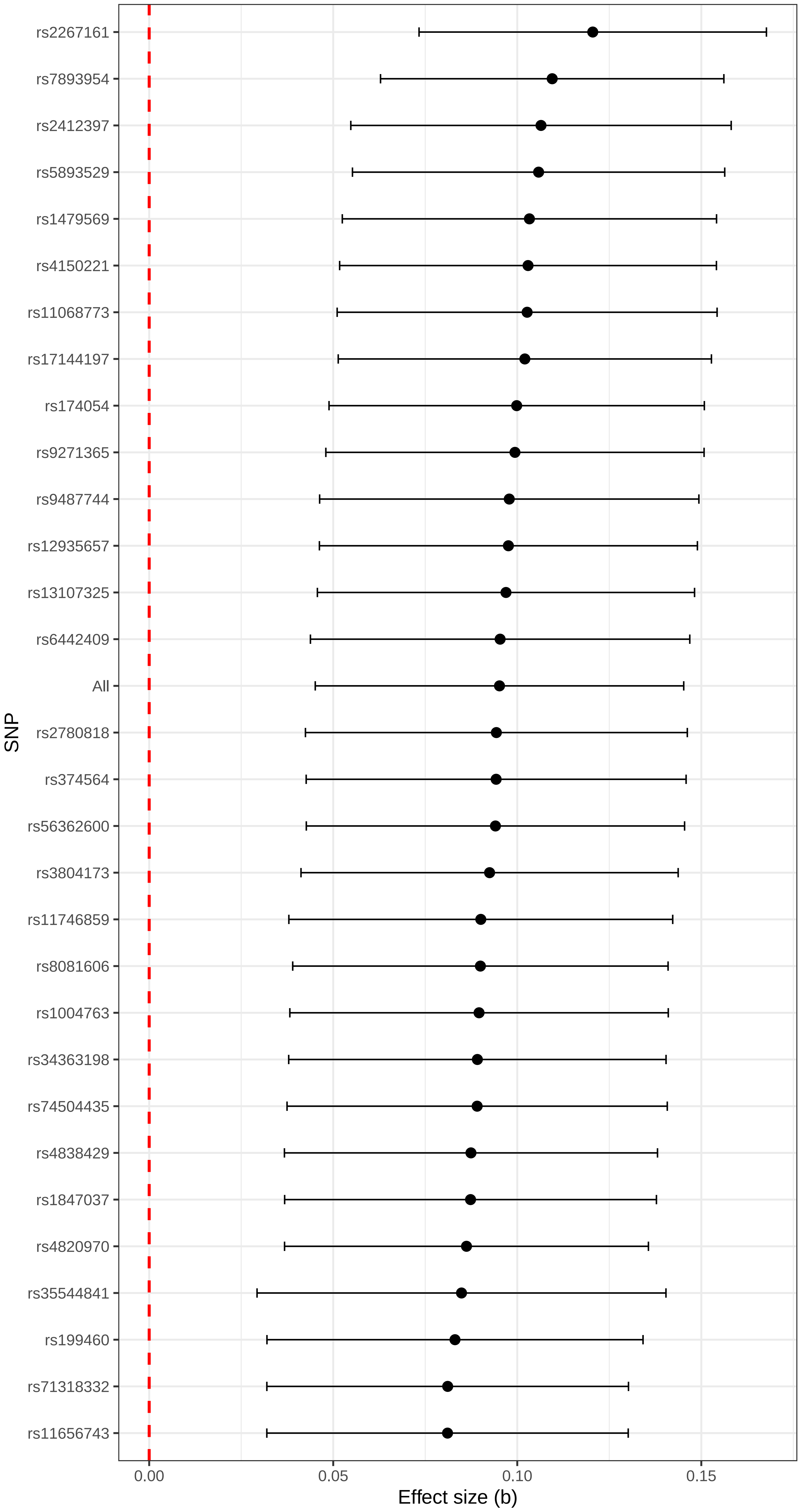
rs2267161
rs7893954
rs2412397
rs5893529
rs1479569
rs4150221
rs11068773
rs17144197
rs174054
rs9271365
rs9487744
rs12935657
rs13107325
rs6442409
All
rs2780818
rs374564
rs56362600
rs3804173
rs11746859
rs8081606
rs1004763
rs34363198
rs74504435
rs4838429
rs1847037
rs4820970
rs35544841
rs199460
rs71318332
rs11656743
SNP
0.00
0.05
0.10
0.15
Effect size (b)
